# Classical Music Students’ Pre-performance Anxiety, Catastrophizing, and Bodily Complaints Vary by Age, Gender, and Instrument and Predict Self-Rated Performance Quality

**DOI:** 10.3389/fpsyg.2022.905680

**Published:** 2022-06-24

**Authors:** Erinë Sokoli, Horst Hildebrandt, Patrick Gomez

**Affiliations:** ^1^Center for Primary Care and Public Health (Unisanté), Department of Occupational and Environmental Health, University of Lausanne, Lausanne, Switzerland; ^2^Swiss University Centre for Music Physiology, Zurich University of the Arts, Zurich, Switzerland; ^3^Swiss University Centre for Music Physiology, Basel University of the Arts, Basel, Switzerland

**Keywords:** music performance anxiety, catastrophizing, bodily complaints, age, gender, musical instrument, musical experience, self-rated musical performance quality

## Abstract

Music performance anxiety (MPA) is a multifaceted phenomenon occurring on a continuum of severity. In this survey study, we investigated to what extent the affective (anxiety), cognitive (catastrophizing), and somatic (bodily complaints) components of MPA prior to solo performances vary as a function of age, gender, instrument group, musical experience, and practice as well as how these MPA components relate to self-rated change in performance quality from practice to public performance. The sample comprised 75 male and 111 female classical music university students, aged 15–45 years. Age was positively associated with anxious feelings and bodily complaints. Compared to male students, female students reported significantly more anxious feelings and catastrophizing. Singers reported less anxious feelings and catastrophizing than instrumentalists. Breathing-, mouth- and throat-related complaints were highest among singers and wind players; hand- and arm-related complaints were highest among string players and pianists. The indices of musical experience and practice had marginal effects. An average of four bodily complaints bothered the participants strongly to very strongly. Worsening in performance quality from practice to public performance was reported by almost half of the participants and was best predicted by anxious feelings and breathing-related complaints. We conclude that age, gender and instrument play a significant role in understanding the phenomenology of MPA. Musicians should be examined according to these characteristics rather than as one homogenous population. In particular, it might be valuable to develop assessment tools for MPA that incorporate items related to the bodily complaints that are most relevant to the different instrument groups. Breathing-related complaints could add an important dimension to the investigation of MPA and music performance. Finally, the high percentage of students reporting worsening of their performance quality from practice to public performance highlights the need of professional support to help music students be able to perform at their best and thrive as artists.

## Introduction

Music performance anxiety (MPA) is one of the leading problems among musicians with potential debilitating effects on musicians’ career and health (Orejudo et al., 2018; Fernholz et al., 2019). MPA is a multifaceted phenomenon that can be understood in terms of affective, cognitive, physiological, and behavioral components (Kenny, 2010). A greater understanding of the predicting factors of MPA has implications not only for theories of MPA but also for its prevention and management and more broadly for teaching and learning. A first aim of this study was to investigate how age, gender, instrument group, and indices of musical experience and practice relate to three facets of MPA: anxious feelings, catastrophizing, and bodily complaints. Anxious feelings refer to the experience of tension, nervousness, apprehension, fear, dread, or panic (Steptoe, 2001). Catastrophizing is a form of worry consisting in the irrational exaggeration of the likelihood of disaster (Steptoe and Fidler, 1987; Liston et al., 2003). Bodily complaints refer to somatic symptoms such as racing heart and dry mouth (Shoup, 1995).

Children as young as three can experience some form of MPA (Boucher and Ryan, 2011), which appears to increase throughout childhood and adolescence (Osborne et al., 2005; Chan, 2011; Patston and Osborne, 2016; Dempsey and Comeau, 2019). Research on the association between age and MPA in adulthood has produced mixed findings. Whereas a few studies suggest that younger musicians may be more affected by MPA than older musicians (Huston, 2001; Osborne and Franklin, 2002; Kenny et al., 2014; Butkovic et al., 2021), others found no significant relationship between age and MPA (Wolfe, 1989; Wesner et al., 1990; van Kemenade et al., 1995; Liston et al., 2003; Kobori et al., 2011; Papageorgi et al., 2013; Dobos et al., 2019; Cohen and Bodner, 2021; Lupiáñez et al., 2021), and one study found a significant positive association between age and MPA in university-level music students (Zarza-Alzugaray et al., 2018).

We could locate approximately 45 studies that investigated gender differences in MPA. In line with the broader literature on anxiety (Bandelow and Michaelis, 2015), about two third of them found female musicians to report significantly higher levels of MPA than male musicians (e.g., Hildebrandt et al., 2012; Casanova et al., 2018; Coskun-Sentürk and Cirakoglu, 2018; Zarza-Alzugaray et al., 2020). About one third of the studies reported no significant gender differences in MPA (e.g., Kobori et al., 2011; Barbar et al., 2014).

With regard to the relationship between instrument and MPA, evidence suggests that (choral) singers may experience lower levels of MPA than (orchestral) instrumentalists (van Kemenade et al., 1995; Schröder and Liebelt, 1999; Sadler and Miller, 2010; Simoens et al., 2015; Robson and Kenny, 2017; Spahn et al., 2021; but see Iusca and Dafinoiu, 2012; Nusseck et al., 2015). Whether different groups of instrumentalists have significantly different levels of MPA is unclear (Fishbein and Middlestadt, 1988; Kenny et al., 2014; Zarza-Alzugaray et al., 2018; Cohen and Bodner, 2021). There is some evidence that instrument groups may differ in the experience of specific bodily complaints (Wolfe, 1989; Studer et al., 2011a), but no in-depth analysis on this issue exists.

Thousands of hours of lessons, practice and performing over many years are necessary to become a professional musician (Ericsson et al., 1993). Researchers have been interested in determining to what extent MPA varies as a function of measures of musical experience and practice. With regard to the amount of practice, studies have either found a significant negative association (Biasutti and Concina, 2014; González et al., 2018; Dobos et al., 2019) or no significant relationship (Kobori et al., 2011; Kenny et al., 2013; Sârbescu and Dorgo, 2014; Lupiáñez et al., 2021; Tan et al., 2021) with MPA. With regard to years studying/playing/performing, the findings are mixed. Studies found that number of years studying/playing/performing was either positively related (Osborne et al., 2005; Patston and Osborne, 2016), negatively related (Huston, 2001; Osborne and Franklin, 2002; Ryan and Andrews, 2009), or more often unrelated (Wolfe, 1989; van Kemenade et al., 1995; Rae and McCambridge, 2004; Sadler and Miller, 2010; Kobori et al., 2011; Kenny et al., 2013; Nusseck et al., 2015; Robson and Kenny, 2017; Casanova et al., 2018; González et al., 2018; Zarza-Alzugaray et al., 2018; Dobos et al., 2019) to MPA. Steptoe and Fidler (1987) found a negative association between years playing in public and MPA in professional orchestral musicians but not in music students and members of an amateur orchestra. With regard to the frequency of performances, the majority of studies suggest that increasing performance frequency is associated with decreasing MPA (Fehm and Schmidt, 2006; Sârbescu and Dorgo, 2014; Simoens et al., 2015; Casanova et al., 2018; Coskun-Sentürk and Cirakoglu, 2018; González et al., 2018; Zarza-Alzugaray et al., 2020; Lupiáñez et al., 2021; but see Huston, 2001; Nusseck et al., 2015). Finally, the literature suggests that music students’ educational level and MPA are not significantly related (Kaspersen and Gotestam, 2002; Liston et al., 2003; Oudejans et al., 2017; Casanova et al., 2018; Lupiáñez et al., 2021).

A second aim of this study was to determine how the MPA components anxious feelings, catastrophizing, and bodily complaints relate to self-rated change in performance quality from practice to public performance. Socially anxious individuals believe that their own abilities fall short of expected audience standards and rate their performance in socially evaluative situations more negatively than socially non-anxious individuals and more poorly than observers do (Rapee and Lim, 1992; Penney and Abbott, 2014). Negative performance appraisal as part of a negative self-appraisal is a main predictor of negative post-event rumination, all of which contribute to maintaining the cycle of social anxiety (Wong and Rapee, 2016). Similar phenomena have been observed among musicians (Osborne and Franklin, 2002; Nielsen et al., 2018). What role does MPA play with regard to the appraisal of one’s own performance? Survey studies have shown that musicians believe that MPA affects their performance quality, with anxious musicians reporting more perceived impairment than non-anxious musicians (Wesner et al., 1990; Clark and Agras, 1991; van Kemenade et al., 1995; Schröder and Liebelt, 1999; Kokotsaki and Davidson, 2003; Fehm and Schmidt, 2006; Papageorgi et al., 2013). A negative association between MPA and either self-rated performance quality, perceived competence or self-reported level of achievement has been reported in studies assessing these concepts separately (Yoshie et al., 2008, 2009b; Chan, 2011; MacIntyre et al., 2012; González et al., 2018; Aufegger and Wasley, 2020). In this study, we wish to extend this line of work by exploring to what extent different facets of MPA predict self-rated change in performance quality from practice to public performance.

Researchers investigating what factors predict or are associated with MPA have used a broad range of tools to measure MPA, from single questions to multi-item questionnaires. However, it remains largely under-researched how age, gender, instrument type, experience, and practice are related to different facets of MPA, and how different facets of MPA are related to self-rated performance quality. A few studies suggest that the strength of these relationships may be dependent on the specific MPA component (Wolfe, 1989; Levy et al., 2011; Sârbescu and Dorgo, 2014; Butkovic et al., 2021; Cornett and Urhan, 2021). For instance, Levy et al. (2011) administered the Performance Anxiety Inventory (Nagel et al., 1981) to 780 world class drum and bugle corps performers and found that females reported more frequent cognitive symptoms than males did. In contrast, there was no significant gender difference for the somatic symptoms.

Solo performances induce higher levels of anxiety than ensemble performances (e.g., Nicholson et al., 2015), and manifestations of MPA before, during and after a performance are different (e.g., Studer et al., 2014; Spahn et al., 2021). If participants refer to different performance settings or different performance phases when reporting on their MPA, interpretation of the results within and between studies is complicated. Moreover, MPA differs as a function of the musical genre (Papageorgi et al., 2013). Contradictory findings in previous research might be partly due to not taking sufficiently into account or controlling for these aspects. In the present study, we control for these factors by investigating the affective, cognitive and somatic facets of MPA in a sample of classical music university students just prior to solo performances.

The first goal of this study was to investigate to what extent age, gender, instrument group, and four indices of musical experience and practice (i.e., academic year, years of instrument study, hours of daily practice, and number of solo performances during the last year) are significant predictors of three facets of MPA, i.e., anxious feelings, catastrophic thinking, and bodily complaints. We expected being female, being an instrumentalist and performing less frequently to be associated with more anxious feelings, catastrophizing, and bodily complaints than being male, being a singer and performing more frequently. Moreover, we hypothesized that instrument group would be a significant predictor of three sub-categories of bodily complaints. Specifically, breathing-related complaints and mouth- and throat-related complaints were expected to be most problematic for singers and wind players, whereas hand- and arm-related complaints were expected to be most problematic for string players and pianists. We predicted academic year to have no significant effects on any MPA components. Given the inconsistency of previous findings, we treated as exploratory issues whether age, years of instrument study and hours of daily practice have significant effects on the MPA components.

The second goal of this study was to investigate whether students’ pre-performance anxious feelings, catastrophic thinking and bodily complaints are significant predictors of their self-rated change in performance quality from practice to public performance. We predicted that all MPA components would be positively associated with a worsening of the performance quality from practice to public performance when tested one by one. Which model would emerge as the best fitting model in multiple regression analysis was treated as an exploratory issue.

## Materials and Methods

### Procedure and Participants

We collected the data presented in this article as part of a questionnaire survey on stage fright in students enrolled at the department of classical music of four universities in the French speaking part of Switzerland. We sent the questionnaire to the students by mail. The study was performed according to the principles of the 1964 Declaration of Helsinki and was approved by the local ethics committee. All students gave their informed written consent to participate. The questionnaire covered several themes, some of which were reported in Studer et al. (2011a,b). As explained in Studer et al. (2011b), we could assume that the sample was representative of the contacted student population.

Participants included in this study were 111 females and 75 males. Their age ranged from 15 to 45 years with a mean of 24.2 (*SD* = 4.3). The sample included 23 singers, 53 wind players, 59 string players, 40 pianists, and 11 percussionists. Four additional students filled in the questionnaire but were excluded from the analyses of this study because their instrument did not belong to one of these five instrument groups. Students’ advancement in their education ranged from the 1st year to the 7th year with the following percentages of students for each year: 1st year: 28%; 2nd year: 29%; 3rd year: 20%; 4th year: 10%; 5th year: 5%; 6th year: 3%; 7th year: 5%. The number of years studying their instrument ranged from 1 to 35 years, with a mean of 13.5 years (*SD* = 4.9). The average number of hours of daily practice ranged from 1 to 10 h with an average of 5 h (*SD* = 1 h and 48 min). Finally, the number of solo performances given in the last 12 months were as follows (percentage of students): 1–5: 38%; 6–10: 38%; 11–15: 11%; 16–20: 6%; 21–25: 1%; 26–30: 1%; 31–35: 1%; >35: 4%.

### Questionnaires

#### Age, Gender, Instrument Group, Musical Experience, and Practice

Participants were asked to indicate their age in years, their gender (male or female), their main instrument and their current academic year since starting university-level education. They further answered the following three questions: (1) “How many years have you been practicing or studying your main instrument (including non-professional and pre-professional studies)?”; (2) “On average, how many hours do you devote to instrumental or vocal practice per day (main instrument and other instruments including personal work, lessons and rehearsals)?”; and (3) “In the last year (last 12 months), how many solo performances have you approximately given (auditions, concerts, exams, competitions, masterclasses, etc., with main instrument and other instruments)?” For the last question, participants had to choose one of the following answers: 1–5, 6–10, 11–15, 16–20, 21–25, 26–30, 31–35, >35. For the analyses, we coded these answers with numbers from 1 to 8.

#### Music Performance Anxiety Components

Participants filled in the following three questionnaires by referring to their experiences just prior to their recent solo performances. We assessed anxious feelings with the 20-item state scale of the State-Trait Anxiety Inventory (Spielberger, 1983; example items are “I feel nervous,” “I feel frightened”). Participants rated each item on a 4-point scale (1 “not at all” to 4 “very much so”). The total score of this questionnaire ranges from 20 (no anxiety) to 80 (extreme anxiety). Following Spielberger’s (1983) instructions, we excluded seven participants with missing values for three or more items. In case of one or two missing values, we replaced them by the mean of the other items and rounded up the sum to the next whole number. Cronbach’s alpha in the present sample was 0.92. We measured catastrophizing with three items originally developed by Steptoe and Fidler (1987). These are “I do not feel in control of this situation; anything might happen,” “I am almost sure to make a dreadful mistake, and that will ruin everything,” and “I do not think I will be able to get through to the end without cracking up.” Participants answered each item on a 5-point scale (0 “never,” 1 “rarely,” 2 “sometimes,” 3 “often,” 4 “very often”). The total score can range from 0 (no catastrophizing) to 12 (extreme catastrophizing). We excluded seven participants with missing values for one or more items. Cronbach’s alpha in the present sample was 0.72. We assessed 29 bodily complaints selected from the Nijmegen Questionnaire (van Dixhoorn and Duivenvoorden, 1985), the Performance Anxiety Questionnaire (Cox and Kenardy, 1993) and interviews conducted with music students. Participants were asked to rate the discomfort associated with each complaint on a 5-point scale (0 “not at all,” 1 “a little,” 2 “moderate,” 3 “strong,” 4 “very strong”). For each participant, we computed a mean score of all bodily complaints. We exclude 12 participants with missing values for three or more bodily complaints. Cronbach’s alpha was 0.86. For each participant, we also computed the number of severe complaints defined as the complaints with a strong or very strong level of discomfort. Finally, we computed mean scores for three sub-categories of bodily complaints: breathing-related complaints (five items), mouth- and throat-related complaints (four items), and hand- and arm-related complaints (five items). We excluded participants with missing values for two or more items (12 for breathing-related complaints; nine for mouth- and throat-related complaints and nine for hand- and arm-related complaints). Cronbach’s alphas for these three sub-categories were 0.74, 0.71, and 0.72, respectively.

#### Self-Rated Change in Performance Quality From Practice to Public Performance

The participants completed the following sentence “When you play/sing in public (compared to when you play/sing alone without an audience), the quality of your performance is generally (a) worse, (b) rather worse, (c) neither worse nor better, (d) rather better, (e) better, (f) I do not know.” Four participants did not answer this question. For the analysis of this variable, we attributed the scores 1 to “better,” 2 to “rather better,” 3 to “neither worse nor better,” 4 to “rather worse,” and 5 to “worse,” so that higher scores correspond to a worsening of the performance quality from practice to public performance.

### Statistical Analyses

We performed all statistical analyses using STATA version 16.1 for Windows (Stata Statistical Software; StataCorp LP, College Station, TX, United States). The alpha level was set at 0.05 for all tests. Where appropriate, we adjusted the *p*-values for multiple comparisons using Tukey’s honestly significant difference (Tukey-Kramer adjustment with unequal sample sizes).

To address the first goal, we regressed scores of anxious feelings, catastrophic thinking and bodily complaints on age, gender, instrument group, and the four indices of musical experience and practice. Age was treated as continuous variable (in year) and gender as categorical variable with female as reference. Instrument was a categorical variable with the five categories singers (reference), wind players, string players, pianists, and percussionists. We treated the four variables about musical experience and practice as continuous variables. In a first step, we performed simple regressions testing the effect of each predictor separately. In a second step, we performed stepwise regression with forward selection with the goal of determining the best fitting model. We used the *p*-values from the simple regressions as criterion to determine which predictor goes in when (starting with the variable with the lowest *p*-value). We used the adjusted *R*^2^ as criterion to keep or drop a variable. The adjusted *R*^2^ increases only if the new term improves the model more than would be expected by chance. It decreases when a predictor improves the model by less than expected by chance. The final model is the one with the highest adjusted *R*^2^ and can include predictors with *p* > 0.05. We computed variance inflation factor (VIF) to evaluate multicollinearity. To test the hypothesis that singers would report lower levels of anxious feelings, catastrophizing, and bodily complaints than the other instrument groups, we contrasted singers vs. the other four groups together.

To address the second goal, we used a similar procedure. We regressed self-rated change in performance quality from practice to public performance (with higher scores corresponding to a worsening of the performance) on the variables anxious feelings, catastrophic thinking, all bodily complaints, breathing-related complaints, mouth- and throat-related complaints, and hand- and arm-related complaints, first one by one and then stepwise with forward selection.

## Results

**FIGURE 1:**
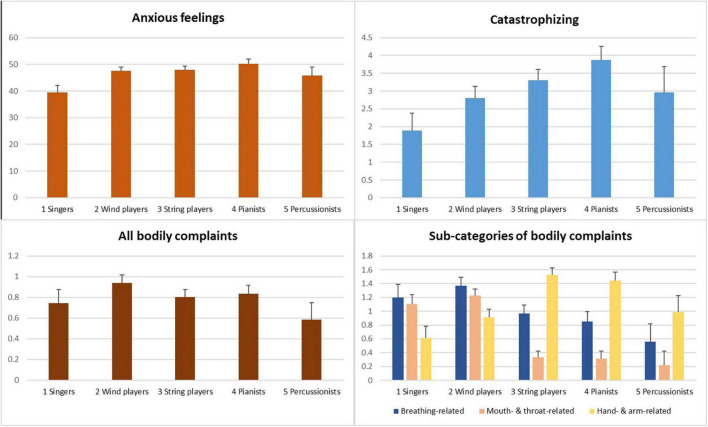
Model-predicted marginal means (*SE*s) of anxious feelings, catastrophizing, all bodily complaints, and sub-categories of bodily complaints for the five instrument groups (see Supplementary Tables 6–8 for all pairwise comparisons).

Descriptive statistics are given in Table 1 and Supplementary Tables 1–5. Figure 1 shows the estimated marginal means of the MPA components for the five instrument groups. Across all tested regression models, VIFs ranged from 1 to 2.61, suggesting no multicollinearity issues (Pallant, 2010).

**TABLE 1:** Descriptive statistics for anxious feelings, catastrophic thinking, bodily complaints, and self-rated change in performance quality from practice to public performance.

	*M*	*SD*	Min	Max	*n*
Anxious feelings	46.8	10.9	25	75	179
Catastrophic thinking	3.1	2.4	0	12	179
All bodily complaints	0.8	0.5	0	2.6	174
Breathing-related complaints	1.1	0.9	0	3.4	174
Mouth- and throat-related complaints	0.7	0.8	0	4	177
Hand- and arm-related complaints	1.2	0.8	0	4	177
Number of severe complaints	4.1	4.1	0	20	174

	***N***	**%**			

Self-rated change in performance quality from practice to public performance					
Better	13	7			
Rather better	38	21			
Neither better nor worse	32	18			
Rather worse	61	33			
Worse	16	9			
I do not know	22	12			

### Effects of Age, Gender, Instrument Group, Musical Experience, and Practice

#### Anxious Feelings

The simple regressions revealed significant effects of age, gender, and number of solo performances (Table 2). Age was positively associated with anxious feelings. Male students reported less anxious feelings than female students. A higher number of solo performances were associated with less anxious feelings. In the multiple regression analysis, we found that the best fitting model was one including age, gender, instrument group, years of instrument study, and number of solo performances, explaining 15.0% of variance in anxious feelings. The effects of age, gender, instrument group, and years of instrument study were statistically significant, whereas the effect of number of solo performances approached significance. As in the simple regression analyses, age was positively associated with anxious feelings, and male students reported less anxious feelings than female students. Singers and pianists exhibited the lowest and highest level of anxious feelings, respectively; the difference between these two groups was significant after adjustment for multiple testing. The contrast singers vs. the other four groups was significant [*F*_(1,167)_ = 8.37, *p* = 0.004, mean difference = 8.40, *SE* = 2.90]. More years of instrument study was significantly associated with lower levels of anxious feelings. A higher number of solo performances tended to be associated with less anxious feelings.

**TABLE 2:** Results of the linear regression analyses for anxious feelings.

	Simple regression						Multiple regression						
Predictors	*B*	*SE*	β	*p*	*R* ^2^	*n*	*B*	*SE*	β	*p*	*R* ^2^	*R* ^2^_adj._	*n*
Age	0.38	0.19	0.15	**0.048**	0.02	179	0.74	0.21	0.29	**<0.001**	0.15	0.11	176
Gender (reference = female)	−3.68	1.64	−0.17	**0.026**	0.03	179	−3.42	1.68	−0.16	**0.044**			
Instrument (reference = singers)				0.16	0.04	179				**0.025**			
Wind players	4.09	2.71	0.17				7.40	2.91	0.34				
String players	5.06	2.67	0.22				7.86	3.12	0.37				
Pianists	6.94	2.85	0.26				10.54	3.24	0.41				
Percussionists	2.07	3.96	0.05				4.81	4.29	0.14				
Academic year	−0.22	0.50	−0.03	0.65	0.00	174							
Years of instrument study	0.06	0.17	0.03	0.72	0.00	179	−0.44	0.21	−0.19	**0.038**			
Hours of daily practice	0.37	0.45	0.06	0.42	0.00	177							
Number of solo performances	−1.14	0.48	−0.18	**0.018**	0.03	176	−0.84	0.49	−0.13	0.089			

*Statistically significant effects are in bold.*

#### Catastrophizing

In the simple regressions, we obtained significant effects of gender and instrument group (Table 3). These two variables also formed the best fitting model in the multiple regression analysis, explaining 9.4% of variance in catastrophizing. Male students reported significantly less catastrophizing than female students. Singers and pianists reported the lowest and highest levels of catastrophizing, respectively; the difference between these two groups was significant after adjustment for multiple testing. The contrast singers vs. the other four groups was significant [*F*_(1,173)_ = 6.13, *p* = 0.014, mean difference = 1.35, *SE* = 0.54].

**TABLE 3:** Results of the linear regression analyses for catastrophic thinking.

	Simple regression						Multiple regression						
Predictors	*B*	*SE*	β	*p*	*R* ^2^	*n*	*B*	*SE*	β	*p*	*R* ^2^	*R* ^2^_adj._	*n*
Age	0.01	0.04	0.03	0.72	0.00	179					0.09	0.07	179
Gender (reference = female)	−0.87	0.36	−0.18	**0.017**	0.03	179	−0.83	0.38	−0.17	**0.028**			
Instrument (reference = singers)				**0.015**	0.07	179				**0.022**			
Wind players	0.64	0.59	0.12				0.92	0.60	0.17				
String players	1.32	0.58	0.26				1.42	0.57	0.28				
Pianists	1.93	0.62	0.33				2.00	0.62	0.34				
Percussionists	0.50	0.86	0.05				1.08	0.23	0.11				
Academic year	0.02	0.11	0.01	0.85	0.00	174							
Years of instrument study	0.02	0.04	0.04	0.56	0.00	179							
Hours of daily practice	0.13	0.10	0.10	0.18	0.01	177							
Number of solo performances	−0.10	0.11	−0.07	0.37	0.00	176							

*Statistically significant effects are in bold.*

#### All Bodily Complaints

The simple regressions revealed significant effects of age and gender (Table 4). Age was positively associated with bodily complaints. Male students reported fewer bodily complaints than female students. In the multiple regression analysis, we found that the best fitting model was one including all seven predictors, explaining 12.6% of variance in bodily complaints. The effect of age was statistically significant, and the effects of gender and number of solo performances approached significance. Increasing age was associated with more bodily complaints. Male students tended to report fewer bodily complaints than female students. A higher number of solo performances tended to be associated with fewer bodily complaints. The contrast singers vs. the other four groups was not significant [*F*_(1,154)_ = 0.10, *p* = 0.75, mean difference = 0.05, *SE* = 0.15].

**TABLE 4:** Results of the linear regression analyses for all bodily complaints.

	Simple regression						Multiple regression						
Predictors	*B*	*SE*	β	*p*	*R* ^2^	*n*	*B*	*SE*	β	*p*	*R* ^2^	*R* ^2^_adj._	*n*
Age	0.02	0.01	0.17	**0.022**	0.03	174	0.03	0.01	0.29	**0.003**	0.13	0.07	165
Gender (reference = female)	−0.16	0.07	−0.16	**0.040**	0.02	174	−0.16	0.08	−0.16	0.054			
Instrument (reference = singers)				0.37	0.02	174				0.28			
Wind players	0.07	0.13	0.07				0.20	0.15	0.18				
String players	−0.05	0.12	−0.05				0.06	0.16	0.06				
Pianists	−0.04	0.13	−0.03				0.09	0.17	0.07				
Percussionists	−0.25	0.19	−0.12				−0.16	0.22	−0.08				
Academic year	−0.01	0.02	−0.04	0.60	0.00	169	−0.01	0.03	−0.05	0.56			
Years of instrument study	−0.00	0.01	−0.04	0.61	0.00	174	−0.02	0.01	−0.16	0.14			
Hours of daily practice	0.00	0.02	0.01	0.89	0.00	173	0.03	0.02	0.11	0.18			
Number of solo performances	−0.04	0.02	−0.12	0.11	0.02	171	−0.04	0.02	−0.15	0.060			

*Statistically significant effects are in bold.*

#### Breathing-Related Complaints

In the simple regression analysis, the effect of instrument group was significant, and the effect of age approached significance (Table 5). In the multiple regression analysis, we found that the best fitting model included age, instrument group, academic year, and number of solo performances. The effects of age and instrument group were significant, and the effect of number of solo performances approached significance. There was a positive association between age and breathing-related complaints. The contrast singers and wind players vs. string players, pianists, and percussionists was significant [*F*_(1,158)_ = 9.39, *p* = 0.003, mean difference = 0.49, *SE* = 0.16]. A higher number of solo performances tended to be associated with fewer breathing-related complaints.

**TABLE 5:** Results of the linear regression analyses for breathing-related complaints.

	Simple regression						Multiple regression						
Predictors	*B*	*SE*	β	*p*	*R* ^2^	*n*	*B*	*SE*	β	*p*	*R* ^2^	*R* ^2^_adj._	*n*
Age	0.03	0.02	0.13	0.098	0.02	174	0.04	0.02	0.17	**0.039**	0.12	0.08	166
Gender (reference = female)	−0.14	0.14	−0.08	0.30	0.01	174							
Instrument (reference = singers)				**0.006**	0.08	174				**0.016**			
Wind players	0.23	0.22	0.12				0.17	0.23	0.08				
String players	−0.22	0.22	−0.12				−0.23	0.23	−0.12				
Pianists	−0.36	0.23	−0.17				−0.35	0.24	−0.16				
Percussionists	−0.58	0.32	−0.16				−0.64	0.32	−0.18				
Academic year	−0.04	0.04	−0.08	0.31	0.01	169	−0.04	0.04	−0.08	0.32			
Years of instrument study	−0.01	0.01	−0.07	0.34	0.01	174							
Hours of daily practice	−0.02	0.04	−0.03	0.67	0.00	173							
Number of solo performances	−0.05	0.04	−0.09	0.23	0.01	171	−0.07	0.04	−0.14	0.076			

*Statistically significant effects are in bold.*

#### Mouth- and Throat-Related Complaints

In the simple regression analysis, we obtained significant effects of instrument group, years of instrument study, and hours of daily practice (Table 6). More years of instrument study and more hours of daily practice were associated with fewer mouth- and throat-related complaints. In the multiple regression analysis, we found that the best fitting model included only instrument group. This model explained 30.1% of the variance in mouth- and throat-related complaints. The contrast singers and wind players vs. string players, pianists, and percussionists was significant [*F*_(1,172)_ = 55.97, *p* < 0.001, mean difference = 0.88, *SE* = 0.12].

**TABLE 6:** Results of the linear regression analyses for mouth- and throat-related complaints.

	Simple regression						Multiple regression						
Predictors	*B*	*SE*	β	*p*	*R* ^2^	*n*	*B*	*SE*	β	*p*	*R* ^2^	*R* ^2^_adj._	*n*
Age	0.02	0.01	0.09	0.21	0.01	177					0.30	0.29	177
Gender (reference = female)	0.04	0.12	0.03	0.71	0.00	177							
Instrument (reference = singers)				**<0.001**	0.30	177				**<0.001**			
Wind players	0.12	0.17	0.07				0.12	0.17	0.07				
String players	−0.77	0.16	−0.46				−0.77	0.16	−0.46				
Pianists	−0.79	0.18	−0.41				−0.79	0.18	−0.41				
Percussionists	−0.88	0.24	−0.27				−0.88	0.24	−0.27				
Academic year	−0.06	0.04	−0.12	0.11	0.02	172							
Years of instrument study	−0.03	0.01	−0.19	**0.010**	0.04	177							
Hours of daily practice	−0.08	0.03	−0.19	**0.010**	0.04	176							
Number of solo performances	0.04	0.04	0.09	0.22	0.01	174							

*Statistically significant effects are in bold.*

#### Hand- and Arm-Related Complaints

In the simple regression analysis, we obtained significant effects of gender, instrument group, hours of daily practice, and number of solo performances (Table 7). Male students reported fewer hand- and arm-related complaints than female students. More hours of daily practice were associated with more hand- and arm-related complaints, and a higher number of solo performances were associated with fewer hand- and arm-related complaints. In the multiple regression analysis, we found that the best fitting model included age, gender, instrument group, hours of daily practice, and number of solo performances. The effects of gender and instrument group were significant, and the effect of number of solo performances approached significance. The contrast string players and pianists vs. singers, wind players and percussionists was significant [*F*_(1,164)_ = 23.91, *p* < 0.001, mean difference = 0.65, *SE* = 0.13].

**TABLE 7:** Results of the linear regression analyses for hand- and arm-related complaints.

	Simple regression						Multiple regression						
Predictors	*B*	*SE*	β	*p*	*R* ^2^	*n*	*B*	*SE*	β	*p*	*R* ^2^	*R* ^2^_adj._	*n*
Age	0.00	0.01	0.01	0.86	0.00	177	0.02	0.01	0.08	0.25	0.26	0.23	173
Gender (reference = female)	−0.35	0.13	−0.21	**0.005**	0.04	177	−0.28	0.12	−0.16	**0.027**			
Instrument (reference = singers)				**<0.001**	0.21	177				**<0.001**			
Wind players	0.30	0.19	0.16				0.30	0.20	0.16				
String players	0.99	0.19	0.56				0.91	0.20	0.51				
Pianists	0.90	0.20	0.44				0.83	0.21	0.40				
Percussionists	0.36	0.28	0.11				0.37	0.31	0.11				
Academic year	−0.00	0.04	−0.00	0.96	0.00	172							
Years of instrument study	0.02	0.01	0.12	0.12	0.01	177							
Hours of daily practice	0.07	0.03	0.16	**0.034**	0.03	176	0.04	0.03	0.08	0.27			
Number of solo performances	−0.10	0.04	−0.20	**0.010**	0.03	174	−0.06	0.04	−0.13	0.079			

*Statistically significant effects are in bold.*

### Music Performance Anxiety Components as Predictors of Self-Rated Change in Performance Quality From Practice to Public Performance

Twenty-two participants answered the question about the change in performance quality from practice to public performance with “I do not know” and were thus not included in the analyses of this variable. The simple regressions revealed that all predictors except mouth- and throat-related complaints were significantly associated with self-rated worsening of performance quality from practice to public performance (Table 8). In the multiple regression analysis, we found that the best fitting model was one including anxious feelings and breathing-related complaints, explaining 12.1% of variance in the outcome variable.

**TABLE 8:** Results of the linear regression analyses for MPA components as predictors of self-rated change in performance quality from practice to public performance.

	Simple regression						Multiple regression						
Predictors	*B*	*SE*	β	*p*	*R* ^2^	*n*	*B*	*SE*	β	*p*	*R* ^2^	*R* ^2^_adj._	*n*
Anxious feelings	0.03	0.01	0.28	**0.001**	0.08	154	0.02	0.01	0.20	**0.017**	0.12	0.11	150
Catastrophizing	0.09	0.04	0.19	**0.021**	0.03	154							
**Bodily complaints**													
All	0.57	0.18	0.24	**0.002**	0.06	151							
Breathing-related	0.38	0.10	0.29	**<0.001**	0.08	151	0.30	0.11	0.23	**0.005**			
Mouth- and throat-related	0.19	0.12	0.13	0.10	0.02	154							
Hand- and arm-related	0.22	0.11	0.16	**0.049**	0.03	153							

*Statistically significant effects are in bold.*

## Discussion

### Predictors of Music Performance Anxiety Components

#### Age

We found that age was positively associated with anxious feelings, all bodily complaints and breathing-related complaints. The age range of our sample was 15–45 years. Our findings are concordant with results by Zarza-Alzugaray et al. (2018) who reported a significant positive association between age and MPA in university music students aged 16–51 years. They are also in agreement with the literature on anxiety disorders according to which the prevalence of anxiety disorders increases until middle age and then decreases in late adulthood (Bandelow and Michaelis, 2015). All the studies that found a significant negative association between age and MPA investigated a population that included musicians in their sixties and seventies, and the samples consisted of professional musicians only (Kenny et al., 2014) or both music students and professionals (Huston, 2001; Osborne and Franklin, 2002; Butkovic et al., 2021). Establishing the link between age and MPA can be difficult because age often covaries with musicians’ professional status. Students and amateur musicians generally suffer from higher MPA than professionals (Steptoe and Fidler, 1987; Kobori et al., 2011; Biasutti and Concina, 2014). Kobori et al. (2011) found that professional status and not age was a significant predictor of MPA. A tendency to less MPA might be seen with an age older than about 45–50 years (Fernholz et al., 2019). Whether this reflects a genuine decrease in MPA after middle age or a cohort effect is unknown. It is possible that only the most resilient musicians stay in the business beyond a certain age whereas those who are more severely affected by MPA leave prematurely.

#### Gender

Female participants reported significantly more anxious feelings, catastrophic thinking, and hand- and arm-related complaints than male participants. We observed a similar trend for all bodily complaints. As mentioned in the introduction, many researchers have observed higher levels of MPA among female musicians than male musicians (e.g., Hildebrandt et al., 2012; Lupiáñez et al., 2021). The present study extends previous work by showing that gender affects all three assessed facets of MPA to a similar degree in a sample of classical music students. The often observed gender effect on MPA is in agreement with the broader literature showing greater vulnerability of females than males for anxiety, worry, and stress (Robichaud et al., 2003; Matud, 2004; Bandelow and Michaelis, 2015). This has been attributed to a combination of gender differences in psychosocial contributors (e.g., childhood sexual abuse, chronic stressors), self-concept, coping styles, genetic, and neurobiological factors (Bangasser et al., 2010; Nolen-Hoeksema, 2012; Bandelow and Domschke, 2015). Among the studies that did not find significant gender differences in MPA (e.g., Fehm and Schmidt, 2006; Khalsa et al., 2009; Kobori et al., 2011; Allen, 2013; Barbar et al., 2014; Cohen and Bodner, 2021), several of them did show a trend in the expected direction. The lack of statistical significance could be due to insufficient statistical power (e.g., Fehm and Schmidt, 2006). On average, the studies that failed to find a significant gender effect on MPA had smaller samples than the studies that found a significant gender effect.

#### Instrument Group

Singers reported significantly lower levels of anxious feelings and catastrophizing than instrumentalists, in particular pianists. This finding is in line and extends the results of several studies acknowledged in the introduction (e.g., Robson and Kenny, 2017). We offer two possible explanations for these results. First, the level of perceived exposure to the audience and thus to negative social evaluation might be lower among singers than instrumentalists. When performing solo, singers are accompanied by a pianist, whereas pianists are on their own and thus the sole object of social evaluation; the other instrumentalists have solo repertoire both with and without accompaniment. Compared to choral ensembles, the number of musicians playing a given instrument or part within instrumental ensembles are generally smaller. Social support provided by non-evaluative others buffers stress responses to performance situations (e.g., Heinrichs et al., 2003). A second possibility is the reliance on an internal instrument for singers as opposed to one that is external for instrumentalists. Ryan and Andrews (2009) argue that singers might feel a greater sense of control over their instrument and thus experience less anxiety than instrumentalists.

Instrument group did not play a significant role in explaining average scores of all bodily complaints but was a major predictor of all three sub-categories of bodily complaints. Singers and wind players reported significantly more severe breathing-related and mouth- and throat-related complaints than the other three groups. String players and pianists reported significantly more severe hand- and arm-related complaints than the other three groups. These results are consistent with an interpretation that the level of bodily discomfort depends on the relevance that specific body parts have for the act of playing the specific instrument. Breathing and the mouth/throat region are particularly important for singing and playing a wind instrument. Superior fine motor skills in the hand/arm region are most important for playing a string instrument or the piano. We speculate that these differences may reflect differences in musicians’ focus of attention.

The finding that the severity of specific bodily complaints strongly depends on the instrument group has the potential to influence the development and use of assessment tools for MPA. Popular questionnaires such as the Performance Anxiety Inventory (Nagel et al., 1981), the Performance Anxiety Questionnaire (Cox and Kenardy, 1993), the Competitive State Anxiety Inventory (Cox et al., 2003), and the Kenny Music Performance Anxiety Inventory (Kenny et al., 2014) include four to seven somatic symptoms. Whereas the symptom “palpitations” appears in all four questionnaires and the symptoms “muscle tension” and “sweaty hands” appear in three of them, breathing-related symptoms are not included in any of them. Important questions to address in future research are whether it would be useful to develop MPA questionnaires specific to each instrument group or an MPA questionnaire that better incorporates items related to the bodily complaints that are most relevant to the different instrument groups.

Knowledge about the associations between instrument group and bodily complaints could also be useful to teachers and therapists in helping students with performance preparation (Hildebrandt, 2009). As an example, one promising approach to improve people’s stress response to demanding tasks is stress arousal reappraisal, which consists in reinterpreting physiological arousal as adaptive and beneficial for task performance (Jamieson et al., 2018). This method could be tailored to the needs of the different instrument groups. For instance, stress arousal reappraisal for singers may be most effective if it mainly focuses on reinterpreting breathing-, mouth-, and throat-related symptoms.

#### Musical Experience and Practice

As hypothesized, participants’ academic year was not a significant predictor of any MPA components. Participants’ number of years of instrument study showed a significant negative relationship with anxious feelings in the multiple regression and with mouth- and throat-related complaints in the simple regression. These findings are in agreement with reports by Huston (2001), Osborne and Franklin (2002), and Ryan and Andrews (2009) and with the idea that length of music training could have a positive impact on MPA. With increasing years of musical experience, musicians may acquire more confidence or develop effective coping strategies to deal with performance stress (Huston, 2001). Nevertheless, we caution against overinterpreting the two significant effects of years of instrument study because the effect on anxious feelings was far from significant in the simple regression, and the effect on mouth- and throat-related complaints was absent in the multiple regression. Moreover, no other significant effects emerged. As reviewed in the introduction, most studies have found no significant association between years of instrument study and MPA (e.g., Casanova et al., 2018).

More hours of daily practice were associated with fewer mouth- and throat-related complaints and more hand- and arm-related complaints, but these effects were not significant in the multiple regressions making us questioning their interpretability. Hours of daily practice had no other significant effects. Overall, our findings are largely in line with reports by Kobori et al. (2011); Kenny et al. (2013), Sârbescu and Dorgo (2014), and Tan et al. (2021) but contrast with studies that reported a significant negative association between amount of practice and MPA (Biasutti and Concina, 2014; González et al., 2018; Dobos et al., 2019). Methodological differences could partly explain these discrepancies. First, there are differences in how authors defined musical practice. Our assessment included individual practice, rehearsal and courses; in contrast, Biasutti and Concina (2014) considered individual practice only. In this regard, Kenny et al. (2013) found a significant negative correlation between MPA and the number of practice sessions per week but not between MPA and the number of hours of weekly practice. Second, the analyses performed in the three studies reporting a significant effect of amount of practice did not include other relevant predictors such as instrument group. In our study, instrument groups significantly differ from each other in their amount of daily practice. Increasing individual practice time is one of the most common strategies that musicians report to use to cope with MPA (Kenny et al., 2014; Burin and Osorio, 2017). Whether practicing long hours reduces MPA is unclear. Practice serves the goal of achieving mastery, which should affect positively MPA as theorized by Wilson (2002). It is possible that beyond a certain number of hours of practice, there is no additional benefit in terms of mastery and MPA. Moreover, an obvious limitation of this measure is that it considers the amount of time practiced but not the quality and type of practice (e.g., Williamon and Valentine, 2000). The degree to which practice is deliberate and reflective might be particularly important (Davis, 2017).

Participants’ number of solo performances tended to be negatively related to anxious feelings, all bodily complaints and two sub-categories of complaints in the multiple regressions. Two of these relationships were statistically significant in the simple regressions. These findings are in line and extend reports by several authors who found that higher number of (solo) public performances are associated with lower levels of MPA (e.g., Casanova et al., 2018; González et al., 2018; Zarza-Alzugaray et al., 2020). The underlying mechanisms of this relationship cannot be inferred from this body of work. Performance experience may allow musicians to improve their ways of managing performance situations and thus, over time, reduce their MPA. Repeated exposure to the object or situation that causes anxiety is part of cognitive-behavioral therapy for anxiety disorders (Bissonnette et al., 2015; Kaczkurkin and Foa, 2015). On the other side, high levels of MPA can lead musicians to avoiding performance situations (e.g., Studer et al., 2011b). Finally, compared to musicians with low levels of MPA, musicians with high levels of MPA may get fewer performance opportunities because of poorer performance.

### Self-Rated Change in Performance Quality From Practice to Public Performance

Of the 160 participants who rated their habitual performance quality, 48% reported that they generally perform rather worse or worse than in practice, whereas 32% reported that they generally perform rather better or better. We are not aware of other survey studies assessing self-rated change in performance quality from practice to public performance. In an experimental study, Studer et al. (2014) found that 44% of music students rated their public performance as being better than their practice performance, whereas 28% rated their practice performance as being better. It is difficult to compare Studer et al.’s (2014) findings with the present findings because in that study all participants performed in the order practice performance-public performance 1 week apart. To what extent the perceived changes in performance quality from practice to public performance corresponds to “objective” changes in performance quality is unknown. In a sample of 101 violin students, 20% performed significantly worse during an examination than during lessons as rated by their violin teachers, but performance changes as rated by the violinists were not assessed (Kivimäki, 1995). Findings have been equivocal regarding the effects of the performance situation (practice vs. public) on expert-rated performance quality (Hamann and Sobaje, 1983; Craske and Craig, 1984; Yoshie et al., 2009a). Correlations between self-ratings and expert ratings are weak to moderate (Kenny et al., 2013; Tief and Gröpel, 2021). The belief that one’s performance is generally worse when performing in public than in practice can contribute to the development and maintenance of a negative self-concept as musician (“I am a poor performer”), with potential adverse career and health consequences. It is important to identify those students who have an unrealistically negative perception of their level of public performance and address their misperception. Studies assessing both self-rated and expert-rated performance quality during practice and public performances are needed (Guyon et al., 2020b).

The predicted positive relationships between MPA components and self-rated worsening of performance quality from practice to public performance were statistically significant for all components except mouth- and throat-related complaints. Breathing-related complaints and anxious feelings together were the best predictors in the multiple regression, explaining 12.1% of the variance. Breathing-related complaints are partly associated with actual changes in breathing patterns. Specifically, shortness of breath and difficulty in breathing deeply enough are positively associated with more sighing and deeper, slower, and more irregular breathing in music students before performing (Guyon et al., 2020a). Breathing (dis)regulation has been associated with cognitive and motor performance (e.g., Karavidas et al., 2010; Grassmann et al., 2016). Respiration is situated at the intersection of automatic functioning and voluntary control and is an important focus of attention of musicians when playing under pressure (Buma et al., 2015; Oudejans et al., 2017). Breathing is a possible target for interventions (van Dixhoorn, 2007; Wells et al., 2012). Based on the findings of the present study and on these considerations, we think that more research is warranted to refine our understanding about the link between breathing-related complaints, breathing patterns, and music performance quality.

### Limitations

The cross-sectional nature of this survey does not allow for any definitive conclusions about causal relationships between variables. Longitudinal studies (e.g., Hildebrandt et al., 2012) and ambulatory assessment studies (e.g., Gomez et al., 2018) measuring musicians’ experiences during practice and different performance situations across time are needed. The focus of the present study was on the “negative side” of performing. It would be important to also consider the “positive side” (e.g., flow, performance boost; Simoens et al., 2015; Cohen and Bodner, 2021). Moreover, we only considered a limited number of potential predictors of the MPA dimensions. Future studies should extend the present work and consider other factors such as personality traits (e.g., Sadler and Miller, 2010). The number of participants in the different instrument groups ranged from 11 to 59. Large differences in sample size between instrument groups is a common problem in research because some instruments are more “popular” than others. Studies have considered woodwind players and brass players separately (e.g., Cohen and Bodner, 2021). Preliminary analyses showed that these two groups behaved very similarly in the present study; we, thus, deemed appropriate to merge them into one. The instrument used to assess self-rated change in performance quality from practice to public performance could be refined by including items that assess different performance criteria and distinguish between factors such as repertoire and performance situations.

## Conclusion

In conclusion, we found that age, gender, and instrument group were the main predictors of MPA components. Older age was associated with more anxious feelings and more bodily complaints; being female and an instrumentalist was associated with more anxious feelings and catastrophizing than being male and a singer. The degree of discomfort associated with breathing-related, mouth- and throat-related, and hand- and arm-related complaints varied significantly across instrument groups. These findings highlight the need to examine musicians according to their age, gender, and instrument group rather than as one homogenous population. Overall, our indices of musical experience and practice played a secondary role in predicting MPA components.

Almost half of the participants reported that the quality of their public performances is generally worse than the quality of their practice performances. Much work is needed to help music students better cope with the “pressure” of performing and ultimately be able to perform at their best and thrive as performing artists. Anxious feelings and breathing-related complaints emerged as the best MPA facets of all to predict self-rated worsening of performance quality from practice to public performance. This finding highlights the potential usefulness of considering breathing-related complaints (which are not part of any of the questionnaires commonly used to assess MPA) in the study of MPA and music performance.

## Data Availability Statement

The raw data supporting the conclusions of this article will be made available by the authors, without undue reservation.

## Ethics Statement

The studies involving human participants were reviewed and approved by the Ethics Committee of the Canton of Vaud, Switzerland. Written informed consent to participate in this study was provided by the participants’ legal guardian/next of kin.

## Author Contributions

All authors listed have made a substantial, direct, and intellectual contribution to the work, and approved it fo publication.

## Conflict of Interest

The authors declare that the research was conducted in the absence of any commercial or financial relationships that could be construed as a potential conflict of interest.

## Publisher’s Note

All claims expressed in this article are solely those of the authors and do not necessarily represent those of their affiliated organizations, or those of the publisher, the editors and the reviewers. Any product that may be evaluated in this article, or claim that may be made by its manufacturer, is not guaranteed or endorsed by the publisher.
