# Supplementary material for: Classical Music Students’ Pre-performance Anxiety, Catastrophizing, and Bodily Complaints Vary by Age, Gender, and Instrument and Predict Self-Rated Performance Quality
Source: Front Psychol. 2022 Jun 24;13:905680. doi: 10.3389/fpsyg.2022.905680 (PMC9263585; doi:10.3389/fpsyg.2022.905680)
Supplement: Supplementary file 1 [file Data_Sheet_1.PDF]

Supplementary Material

Table S1. Pearson correlation matrix.

	1	2	3	4	5	6	7	8	9	10	11	12
1. Age												
2.Gender	-0.03											
3. Academic year	0.34*	-0.02										
4. Years of instrument study	0.36*	0.04	0.38*									
5. Hours of daily practice	0.01	0.09	0.04	0.21*								
6. Number of solo performances	0.10	-0.00	0.15	0.06	0.06							
7. Anxious feelings	0.15*	-0.17*	-0.03	0.03	0.06	-0.18*						
8. Catastrophic thinking	0.03	-0.18*	0.01	0.04	0.10	-0.07	0.61*					
9. All bodily complaints	0.17*	-0.16*	-0.04	-0.04	0.01	-0.12	0.43*	0.35*				
10. Breathing-related complaints	0.13	-0.08	-0.08	-0.07	-0.03	-0.09	0.30*	0.25*	0.77*			
11. Mouth- and throat-related complaints	0.09	0.03	-0.12	-0.19*	-0.19*	0.09	0.06	0.09	0.58*	0.48*		
12. Hand- and arm-related complaints	0.01	-0.21*	-0.00	0.12	0.16*	-0.20*	0.37*	0.27*	0.56*	0.25*	-0.09	
13. Self-rated change in performance quality	-0.04	-0.09	-0.10	0.03	-0.07	-0.03	0.28*	0.19*	0.24*	0.29*	0.13	0.16*

Note: For gender female = 0 and male = 1. * $p < .05$. The variable self-rated change in performance quality from practice to public performance was scored so that higher scores correspond to a worsening of the performance quality from practice to public performance.

Table S2. Statistics for male and female participants (means and *SD*s in parentheses).

	Males	Females	Statistics
N	75	111	
Age	24.0 (4.3)	24.3 (4.2)	*t*(184) = 0.45, *p* = .66, *d* = 0.07
Instrument groups			**chi^2(4) = 22.95, *p*<.001**
Singers	5	18	
Wind players	29	24	
String players	19	40	
Pianists	12	28	
Percussionists	10	1	
Academic year	2.6 (1.7)	2.7 (1.6)	*t*(179) = 0.29, *p* = .77, *d* = 0.04
Years of instrument study	13.7 (4.2)	13.3 (5.3)	*t*(183) = -0.48, *p* = .64, *d* = 0.07
Hours of daily practice	5.1 (1.9)	4.8 (1.7)	*t*(181) = -1.18, *p* = .24, *d* = 0.18
Number of solo performances	2.2 (1.8)	2.2 (1.6)	*t*(180) = 0.06, *p* = .95, *d* = 0.01
Anxious feelings	44.6 (10.4)	48.3 (11.0)	***t*(177) = 2.25, *p* = .026, *d* = 0.34**
Catastrophic thinking	2.6 (2.0)	3.4 (2.6)	***t*(177) = 2.42, *p* = .017, *d* = 0.37**
All bodily complaints	0.7 (0.5)	0.9 (0.5)	***t*(172) = 2.07, *p* = .040, *d* = 0.32**
Breathing-related complaints	1.0 (0.9)	1.1 (0.9)	*t*(172) = 1.03, *p* = .30, *d* = 0.16
Mouth- and throat-related complaints	0.7 (0.9)	0.7 (0.7)	*t*(175) = -0.37, *p* = .71, *d* = 0.06
Hand- and arm-related complaints	1.0 (0.8)	1.3 (0.8)	***t*(175) = 2.82, *p* = .005, *d* = 0.43**

Note: Number of solo performances is on a scale from 1 to 8 with 1 = 1-5 performances and 8 = more than 35 performances. Statistically significant effects are in bold.

Table S3. Statistics for the five instrument groups (means and *SD*s in parentheses).

	Singers	Wind players	String players	Pianists	Percussionists	Statistics
N	23	53	59	40	11	
Age	25.7 (4.6)	24.4 (3.8)	23.4 (4.4)	24.4 (4.7)	23.9 (2.5)	$F(4,181) = 1.34$ $p = .26$
Gender (% male)	22	55	32	30	91	Fisher's exact $\boldsymbol{p}$**<.001**
Academic year	2.5 (1.4)	2.5 (1.6)	2.6 (1.7)	3.1 (1.7)	2.7 (1.9)	$F(4,176) = 0.82$ $p = .45$
Years of instrument study	8.5 (3.8)	12.7 (3.8)	14.7 (4.8)	15.7 (5.0)	13.1 (4.1)	$F(4,180) = 11.33$ $\boldsymbol{p}$**<.001**
Hours of daily practice	4.0 (1.9)	4.4 (1.7)	5.2 (1.6)	5.4 (1.7)	6.7 (1.5)	$F(4,178) = 7.00$ $\boldsymbol{p}$**<.001**
Number of solo performances	3.4 (2.3)	2.2 (1.6)	1.8 (1.1)	2.2 (1.9)	1.6 (0.8)	$F(4,177) = 4.44$ $\boldsymbol{p}$ **= .002**
Anxious feelings	42.5 (10.0)	46.6 (11.5)	47.5 (9.2)	49.4 (12.2)	44.5 (11.3)	$F(4,174) = 1.67$ $p = .16$
Catastrophic thinking	2.0 (2.0)	2.7 (2.2)	3.4 (2.6)	4.0 (2.5)	2.5 (1.6)	$F(4,174) = 3.18$ $\boldsymbol{p}$ **= .015**
All bodily complaints	0.8 (0.6)	0.9 (0.5)	0.8 (0.5)	0.8 (0.5)	0.6 (0.3)	$F(4,169) = 1.07$ $p = .37$
Breathing-related complaints	1.2 (0.8)	1.4 (1.0)	1.0 (0.8)	0.8 (0.8)	0.6 (0.5)	$F(4,169) = 3.80$ $\boldsymbol{p}$ **= .006**
Mouth- and throat-related complaints	1.1 (0.9)	1.2 (0.9)	0.3 (0.5)	0.3 (0.4)	0.2 (0.4)	$F(4,172) = 18.58$ $\boldsymbol{p}$**<.001**
Hand- and arm-related complaints	0.6 (0.5)	0.9 (0.6)	1.6 (0.8)	1.5 (0.9)	1.0 (0.5)	$F(4,172) = 11.19$ $\boldsymbol{p}$**<.001**

Note: Number of solo performances is on a scale from 1 to 8 with 1 = 1-5 performances and 8 = more than 35 performances. Statistically significant effects are in bold.

Table S4. Descriptive statistics (means and *SD*s in parentheses) for the 29 bodily complaints for all participants and the five instrument groups.

	All participants	Singers	Wind players	String players	Pianists	Percussionists
Shaking hands	**1.84** (1.38)	1.13 (1.39)	**1.52** (1.30)	**2.20** (1.35)	**2.11** (1.39)	**2.00** (1.10)
Palpitations	**1.59** (1.28)	**1.65** (1.30)	**1.63** (1.31)	**1.49** (1.24)	**1.75** (1.44)	**1.27** (0.79)
Cold hands	**1.43** (1.31)	0.83 (1.03)	0.82 (0.99)	**1.91** (1.30)	**2.08** (1.34)	0.82 (1.08)
Sweaty hands	**1.35** (1.21)	0.36 (0.58)	1.18 (1.01)	**1.83** (1.22)	**1.53** (1.37)	**1.09** (1.04)
Unable to breathe deeply	**1.26** (1.39)	**1.50** (1.41)	**1.81** (1.56)	1.07 (1.31)	0.82 (1.18)	0.90 (0.88)
Faster or deeper breathing	1.15 (1.15)	1.39 (1.23)	1.24 (1.23)	1.14 (1.17)	0.97 (1.01)	0.91 (0.94)
Muscle tension	1.14 (1.23)	**1.48** (1.27)	0.90 (1.19)	1.27 (1.26)	1.11 (1.20)	0.90 (1.29)
Shortness of breath	1.11 (1.33)	**1.52 (**1.21)	**1.84** (1.49)	0.71 (1.06)	0.80 (1.23)	0.09 (0.30)
Sweating	1.10 (1.13)	0.70 (0.97)	1.14 (1.06)	**1.29** (1.20)	1.00 (1.19)	**1.09** (1.14)
Dry mouth	1.07 (1.29)	**2.05** (1.40)	**2.04** (1.34)	0.41 (0.74)	0.39 (0.64)	0.27 (0.47)
Tight feelings in chest	1.02 (1.21)	1.14 (1.17)	1.17 (1.37)	1.05 (1.06)	0.89 (1.30)	0.45 (0.93)
Fatigue	0.97 (1.25)	1.09 (1.24)	1.02 (1.41)	0.89 (1.23)	0.89 (1.03)	**1.18** (1.40)
Stiff fingers and arms	0.86 (1.23)	0.39 (0.78)	0.54 (0.94)	1.19 (1.36)	**1.16** (1.50)	0.55 (0.82)
Confusion/loss of contact with reality	0.79 (1.21)	0.61 (1.20)	0.83 (1.28)	0.70 (1.08)	1.05 (1.37)	0.55 (0.93)
Stomach pain/abdominal cramps	0.78 (1.12)	0.87 (1.25)	0.84 (1.20)	0.57 (0.89)	0.92 (1.23)	0.90 (1.10)
Frequent need to urinate	0.75 (1.00)	0.87 (0.92)	0.90 (1.20)	0.68 (0.97)	0.71 (0.90)	0.27 (0.47)

Table S4 (continued).

	All participants	Singers	Wind players	String players	Pianists	Percussionists
Bloated feeling in stomach	0.73 (1.04)	0.87 (1.32)	0.71 (0.87)	0.60 (0.98)	0.92 (1.20)	0.60 (0.84)
Forgetting to breathe/apnea	0.73 (1.18)	0.30 (0.70)	0.88 (1.32)	0.84 (1.19)	0.62 (1.14)	0.73 (1.42)
Diarrhea or constipation	0.63 (0.96)	0.96 (1.07)	0.65 (1.05)	0.44 (0.79)	0.74 (1.03)	0.40 (0.70)
Tightness in the throat	0.56 (0.99)	0.87 (1.10)	0.86 (1.24)	0.35 (0.77)	0.47 (0.86)	0.00 (0.00)
Tightness of the lips	0.53 (1.04)	0.70 (1.15)	1.27 (1.43)	0.18 (0.43)	0.18 (0.56)	0.00 (0.00)
Modified swallowing	0.51 (0.98)	0.91 (1.24)	0.67 (1.21)	0.40 (0.81)	0.22 (0.42)	0.45 (1.04)
Tingling fingers	0.48 (0.98)	0.17 (0.39)	0.27 (0.70)	0.74 (1.20)	0.63 (1.13)	0.27 (0.65)
Dizziness	0.32 (0.80)	0.27 (0.63)	0.15 (0.51)	0.46 (0.96)	0.42 (0.97)	0.09 (0.30)
Headache	0.28 (0.65)	0.30 (0.56)	0.24 (0.59)	0.18 (0.51)	0.47 (0.89)	0.18 (0.60)
Blurred vision	0.27 (0.69)	0.13 (0.34)	0.42 (1.01)	0.23 (0.54)	0.27 (0.65)	0.09 (0.30)
Chest pain	0.25 (0.62)	0.30 (0.63)	0.39 (0.86)	0.25 (0.54)	0.13 (0.34)	0.00 (0.00)
Voice modification	0.23 (0.67)	0.86 (1.32)	0.17 (0.48)	0.14 (0.44)	0.14 (0.48)	0.00 (0.00)
Vomiting	0.21 (0.70)	0.22 (0.85)	0.28 (0.76)	0.11 (0.45)	0.26 (0.79)	0.27 (0.90)

Note: Breathing-related complaints are “unable to breathe deeply”, “faster or deeper breathing”, “shortness of breath”, “tight feelings in the chest” and “forgetting to breathe/apnea”. Mouth- and throat-related complaints are “dry mouth”, tightness in the throat”, “tightness of the lips” and “modified swallowing”. Hand- and arm-related complaints are “shaking hands”, “cold hands”, “sweaty hands”, “stiff fingers and arms” and “tingling fingers”. The top five bodily complaints in each column are in bold.

Table S5. Frequencies for the 29 bodily complaints listed from the highest to the lowest mean score across all participants.

	Degree of discomfort					
	N	Not at all (%)	A little (%)	Moderate (%)	Strong (%)	Very strong (%)
Shaking hands	178	24	19	21	22	14
Palpitations	175	26	25	24	17	9
Cold hands	176	35	18	24	16	7
Sweaty hands	174	33	23	26	13	5
Unable to breathe deeply	174	46	13	19	13	9
Faster or deeper breathing	173	39	25	19	16	1
Muscle tension	175	43	21	18	13	5
Shortness of breath	172	51	15	14	15	6
Sweating	177	40	28	20	10	3
Dry mouth	175	49	21	13	11	7
Tight feelings in chest	173	49	20	17	11	4
Fatigue	177	51	21	13	8	6
Stiff fingers and arms	176	58	18	10	9	6
Confusion/loss of contact with reality	175	62	14	11	7	5
Stomach pain/abdominal cramps	176	59	19	11	9	3
Frequent need to urinate	179	55	22	17	2	3

Table S5 (continued).

		Degree of discomfort				
	N	Not at all (%)	A little (%)	Moderate (%)	Strong (%)	Very strong (%)
Bloated feeling in stomach	175	58	21	11	7	2
Forgetting to breathe/apnea	177	68	6	12	10	3
Diarrhea or constipation	177	63	20	11	6	1
Tightness in the throat	177	69	15	10	5	2
Tightness of the lips	176	73	11	7	6	3
Modified swallowing	174	72	15	6	5	2
Tingling fingers	178	76	8	10	4	2
Dizziness	173	83	8	5	3	1
Headache	177	82	10	7	1	0
Blurred vision	176	83	11	4	1	1
Chest pain	178	82	12	4	1	1
Voice modification	174	87	6	5	2	1
Vomiting	178	89	4	3	2	1

Note: Breathing-related complaints are “unable to breathe deeply”, “faster or deeper breathing”, “shortness of breath”, “tight feelings in the chest” and “forgetting to breathe/apnea”. Mouth- and throat-related complaints are “dry mouth”, tightness in the throat”, “tightness of the lips” and “modified swallowing”. Hand- and arm-related complaints are “shaking hands”, “cold hands”, “sweaty hands”, “stiff fingers and arms” and “tingling fingers”.

Table S6. Pairwise comparisons between instrument groups with Tukey adjustment (significant effects in bold) for anxious feelings (below the diagonal) and catastrophizing (above the diagonal). See Figure 1 for the model-predicted marginal means.

	Singers	Wind players	String players	Pianists	Percussionists
Singers		.54	.10	**.012**	.74
Wind players	.052		.80	.22	.99
String players	.056	.99		.77	.99
Pianists	**.010**	.78	.85		.80
Percussionists	.57	.98	.97	.75	

Table S7. Pairwise comparisons between instrument groups with Tukey adjustment (significant effects in bold) for breathing-related complaints (below the diagonal) and mouth- and throat-related complaints (above the diagonal). See Figure 1 for the model-predicted marginal means.

	Singers	Wind players	String players	Pianists	Percussionists
Singers		.95	**<.001**	**<.001**	**.003**
Wind players	.95		**<.001**	**<.001**	**<.001**
String players	.85	.15		.99	.99
Pianists	.60	.062	.97		.99
Percussionists	.28	**.041**	.59	.86	

Table S8. Pairwise comparisons between instrument groups with Tukey adjustment (significant effects in bold) for hand- and arm-related complaints. See Figure 1 for the model-predicted marginal means.

	Singers	Wind players	String players	Pianists	Percussionists
Singers					
Wind players	.57				
String players	**<.001**	**.001**			
Pianists	**.001**	**.023**	.98		
Percussionists	.76	.99	.23	.45	
